# Unveiling the dual role of heterotrophic ammonia-oxidizing bacteria: enhancing plant regrowth through modulating cytokinin delivery

**DOI:** 10.3389/fmicb.2023.1268442

**Published:** 2023-09-20

**Authors:** Xiao-Ling Wang, Zhen-Qiang Si, Hao Yu, Lin Qi, Wei Liu, Jiang Shi, Peng Song

**Affiliations:** College of Agriculture, Henan University of Science and Technology, Luoyang, Henan, China

**Keywords:** ammonia oxidizing bacteria, Italian ryegrass, regrowth, cytokinin, soil nitrification

## Abstract

This study aims to investigate the dual impacts of heterotrophic ammonia-oxidizing bacteria (HAOB) strains on the regrowth of Italian ryegrass by studying cytokinin delivery from roots to leaves. The dual impacts encompass both the “soil-inside-role” and “soil-outside-role,” which refer to the HAOB operating inside and outside the rhizosphere soil within the rhizosphere microenvironment. The experimental design consisted of two sets of experiments, Exp-1 and Exp-2, involving different treatments. In Exp-1, various concentrations of NO_3_^−^ were added to the roots to observe the soil-inside-role on cytokinin delivery from roots to leaves. In Exp-2, NO_3_^−^ addition was combined with HAOB inoculation to observe the combined effects of the root-outside-role and root-inside-role on cytokinin synthesis and transport. The results indicated that NO_3_^−^ concentrations ranging from 30 to 40 mmol L^−1^ had the most optimal effect on increasing leaf cytokinin content and delivery from roots to leaves, consequently promoting greater leaf regrowth biomass. When inoculated, the HAOB strain significantly increased rhizosphere soil nitrification rates under the soil-inside-role, leading to increased NO_3_^−^ release from the soil and a subsequent boost in cytokinin delivery from roots to leaves. Additionally, the HAOB strain independently enhanced cytokinin delivery from roots to leaves outside the rhizosphere soil within the rhizosphere microenvironment, demonstrating its soil-outside-role. The combined effects of the soil-inside-role and soil-outside-role substantially increased leaf cytokinin content, playing a crucial role in promoting Italian ryegrass regrowth. The study’s findings shed light on the mechanisms through which HAOB can enhance plant growth by performing dual roles in the rhizosphere, offering potential applications in agriculture. Understanding the interaction between HAOB, cytokinins, and plant growth could lead to more effective strategies for improving crop productivity and promoting sustainable agriculture.

## 1. Introduction

Plant growth promoting rhizobacteria (PGPR) represent a diverse group of bacteria that colonize the rhizosphere. The rhizosphere, encompassing the soil surrounding the roots (rhizosphere soil), the root surface (rhizoplane), and the internal tissues of the roots (endorhizosphere), constitutes a specialized and confined environment (McNear, 2013; Kwak et al., 2018). PGPR play a pivotal role in enhancing plant growth and have gained recognition in agriculture. They offer various advantages, including improved drought stress tolerance, facilitation of nitrogen nutrient cycling, and stimulation of plant hormone synthesis (Forni et al., 2019; Arkhipova et al., 2020; Naitam and Kaushik, 2021). Investigating the mechanisms underlying the positive influence of PGPR on plant growth can significantly contribute to the agricultural industry.

In the rhizosphere, PGPR colonize two primary sites: the rhizosphere soil and various parts of the roots, including the rhizoplane and endorhizosphere. Certain PGPR primarily operate within the rhizosphere soil, promoting plant growth by enhancing nutrient release (Borgi et al., 2020; Wang et al., 2021). Others predominantly function within the internal root tissues, stimulating plant growth through hormone synthesis and the production of other growth-promoting substances (Luo et al., 2019; Nascimento et al., 2020). These effects on plant growth in different colonization sites within rhizosphere microenvironment are referred to as the “soil-inside-role” and “soil-outside-role,” respectively, as they operate inside and outside the rhizosphere soil. While no reports currently exist of a bacterium simultaneously performing these two roles in diverse rhizosphere environments, an investigation into the functions of PGPR across various rhizosphere regions may reveal the existence of such dual-function bacteria. This discovery would enhance our understanding of PGPR’s impact on plant growth and provide more effective strategies for agricultural applications.

Soil ammonia-oxidizing bacteria play a pivotal role in promoting plant growth by facilitating root cytokinin synthesis. As the primary bacteria involved in soil nitrification, ammonia-oxidizing bacteria accelerate this process, leading to increased release of soil nitrate (NO_3_^−^). Soil NO_3_^−^, in turn, triggers cytokinin synthesis in plant roots, thereby influencing overall plant growth (Landrein et al., 2018; Poitout et al., 2018; Wang et al., 2018). In a recent study (Wu et al., 2022), a heterotrophic ammonia-oxidizing bacteria (HAOB) strain exhibited a strong correlation with the transport of cytokinins from roots to leaves. This correlation resulted in an elevation of leaf cytokinin content, subsequently contributing to the regrowth of Italian ryegrass following defoliation. Additionally, it is worth noting that other pathways may also contribute to the process of how PGPR enhance growth through cytokinins, given the complexity of the mechanism involved. For instance, certain rhizobia and *Bacillus* species have been shown to regulate cytokinin synthesis by entering plant roots (Han et al., 2023). Nevertheless, limited information is available on whether HAOB can simultaneously enhance cytokinin synthesis through multiple pathways.

The study aimed to investigate the effects of a HAOB strain on cytokinin synthesis and transport, encompassing both its soil-inside-role and soil-outside-role. The hypothesis was that the strain would influence cytokinin levels and distribution within the plant through these two roles. Confirming this hypothesis would reveal a novel mechanism through which soil microorganisms impact plant growth. Ammonia-oxidizing bacteria plays a crucial role in the nitrogen cycle, making it indispensable for agriculture. Italian ryegrass was selected as the test plant for its rapid growth and the potential for observable growth variations attributed to root-originated cytokinins, and it’s widely planted worldwide, benefiting the grassland industry.

To test the hypothesis, an excess of NO_3_^−^ was added to roots to observe the soil-inside-role of HAOB strain on cytokinin delivery from roots to leaves. The disparity in the cytokinin delivery rates from roots to leaves between AOB-induced and soil-inside-role indicated soil-outside-role effects. Additionally, leaf cytokinin and its transport were also assessed to comprehend the impact of the root-outside-role and root-inside-role on Italian ryegrass regrowth.

## 2. Materials and methods

### 2.1. Experimental design

In this study, the test bacterium used was the HAOB strain S2_8_1, which belongs to the *Ensifer* genus and is assigned the GenBank accession number ON667919. This strain is stored at the Chinese Center for Preservation of Typical Cultures (Wuhan University, Wuhan, China) with deposit number CCTCC NO: M2021374.

A pot experiment was conducted at Henan University of Science and Technology in Luoyang City, Henan Province, China. The site receives an average annual rainfall of 601 mm and has an average temperature of 14.2°C. Italian ryegrass cv. Barwoltra (Barenbrug, China) was chosen as the test grass. In early March 2022, pots with dimensions 20 cm (diameter) × 25 cm (height) and a volume of 13.5 L were prepared. Each pot was filled with 5.8 kg of brown soil, containing 23.7 g kg^−1^ of organic carbon, 2.2 g kg^−1^ of total nitrogen, and 10.5 mg kg^−1^ of available phosphorus.”

Ryegrass seeds were planted in the prepared pots and cultivated in a greenhouse at a constant temperature of 24°C for a duration of 2 weeks. Subsequently, six seedlings displaying consistent growth were carefully transplanted into 200 plastic pots. The pots were then placed in sunlight, allowing the seedlings to thrive over a period of 6 weeks. For experiment-1 (Exp-1) and experiment-2 (Exp-2), 63 and 54 pots, respectively, were selected. These pots contained uniformly and vigorously growing seedlings, and adequate water supply and light were ensured to minimize experimental errors. Exp-1 involved the addition of NO_3_^−^ to the roots, while Exp-2 involved both the addition of NO_3_^−^ to the roots and the inoculation of the heterotrophic AOB strain to the roots.

#### 2.1.1. Experiment-1 (Exp-1)

**Figure 1:**
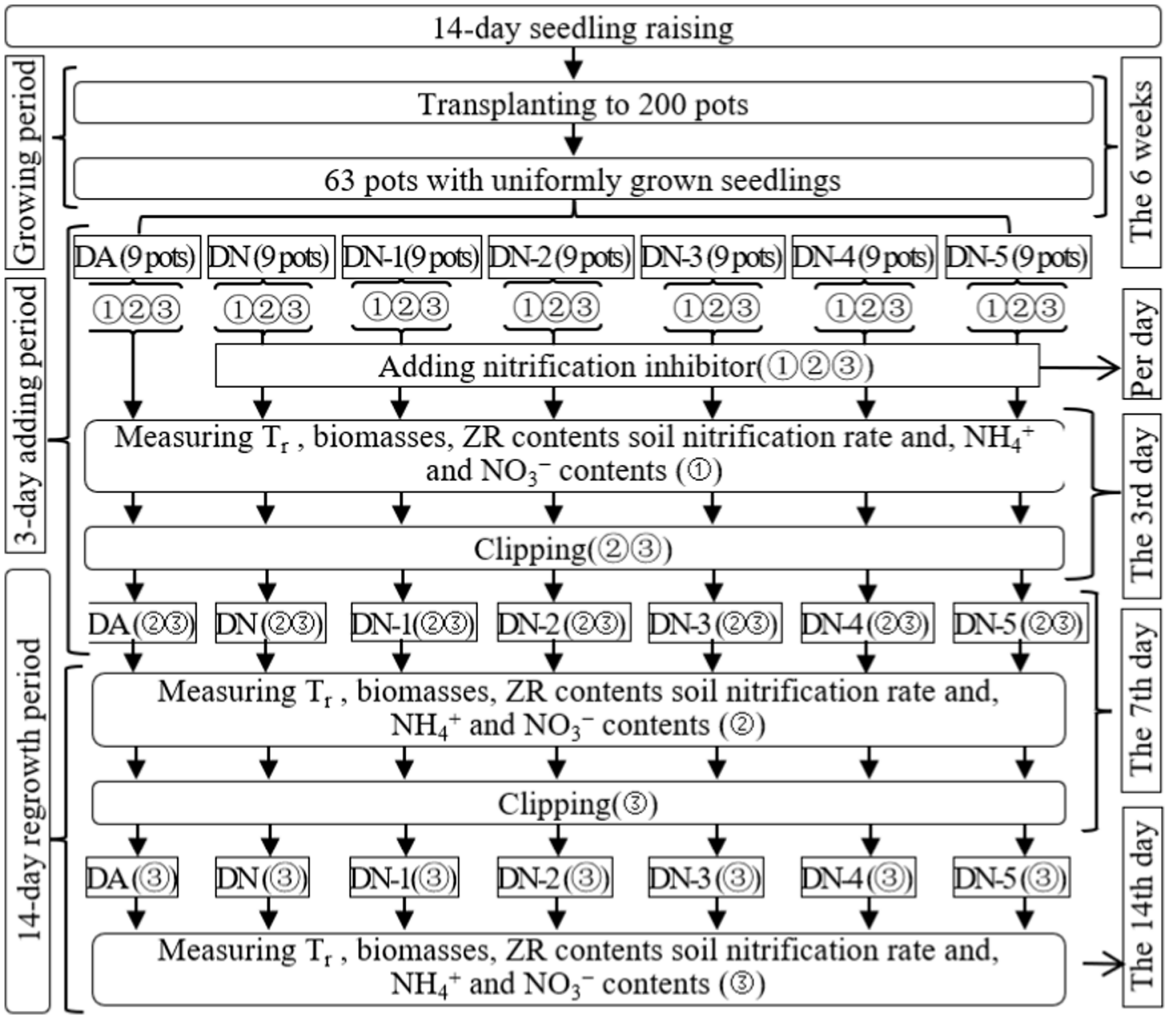
Schematic diagram for the experimental design of experiment-1. DA, DN, DN1, DN2, DN3, DN4 and DN5 represent conventional water supply treatment, adding DMPP, adding DMPP and 10 mmol L^−1^ NO_3_^−^-N, adding DMPP and 20 mmol L^−1^ NO_3_^−^-N, adding DMPP and 30 mmol L^−1^ NO_3_^−^-N, adding DMPP and 40 mmol L^−1^ NO_3_^−^-N and adding DMPP and 50 mmol L^−1^ NO_3_^−^-N, respectively. “①,” “②,” and “③” show the first, second, and third subgroups of each treatment, respectively. The 3rd days refer to the 3rd days of the 3 days addition period. The 7th and 14th days refer to the 14 days regrowth period. All indexes refer to all the indexes that were measured in the present study. *T*_r_ represents the transpiration rate, respectively.

In Exp-1, the 63 selected pots were divided into seven groups, each consisting of nine pots corresponding to various treatments in the study. The treatments included: (1) regrowth (DA), (2) regrowth with the addition of a nitrification inhibitor (DN), (3) regrowth with the addition of a nitrification inhibitor and 10 mmol L^−1^ of NO_3_^−^-N (DN-1), (4) regrowth with the addition of a nitrification inhibitor and 20 mmol L^−1^ of NO_3_^−^-N (DN-2), (5) regrowth with the addition of a nitrification inhibitor and 30 mmol L^−1^ of NO_3_^−^-N (DN-3), (6) regrowth with the addition of a nitrification inhibitor and 40 mmol L^−1^ of NO_3_^−^-N (DN-4), (7) regrowth with the addition of a nitrification inhibitor and 50 mmol L^−1^ of NO_3_^−^-N (DN-5). Figure 1 provides a clear representation of the trial timeline, treatment configurations, and the associated parameters measured across the 63 pots.

In Exp-1, a 3 days period involving the daily addition of approximately 30 mL of a 1.5 g L^−1^ DMPP aqueous solution was established, following by a 14 days regrowth period. This addition aimed to inhibit soil nitrification during the regrowth phase and was applied to all treatments except DA. This approach was based on previous research, which indicated a lag time of approximately 3 days for the inhibitory effect of DMPP on Italian ryegrass regrowth (Wu et al., 2022). Throughout the regrowth period, soil water content was maintained at 65–70% of field capacity by adding either NO_3_^−^ solutions or water to the treatments. The treatments of DN-1, DN-2, DN-3, DN-4, and DN-5 received NO_3_^−^ solutions with concentrations of 10, 20, 30, 40, and 50 mmol L^−1^, respectively, while DA and DN received water. Applications of NO_3_^−^ solutions or water were made when the soil water content dropped below 65% of field capacity. Soil water content was calculated using Formula (1) as described by Wang et al. (2018).


(1) $$SWC=\frac{B_{t}-B_{d}-B_{e}-B_{p}}{B_{d}\times FWC}\times 100\%$$

where SWC is the soil water content, *B*_t_ is the temporary whole pot weight, *B*_d_ is the net dried soil weight, *B*_e_ is the empty pot weight, *B*_p_ is the estimated fresh weight of all plants, and FWC is the field water capacity in each pot, respectively. *B*_p_ was determined on extra pots early.

#### 2.1.2. Experiment-2 (Exp-2)

**Figure 2:**
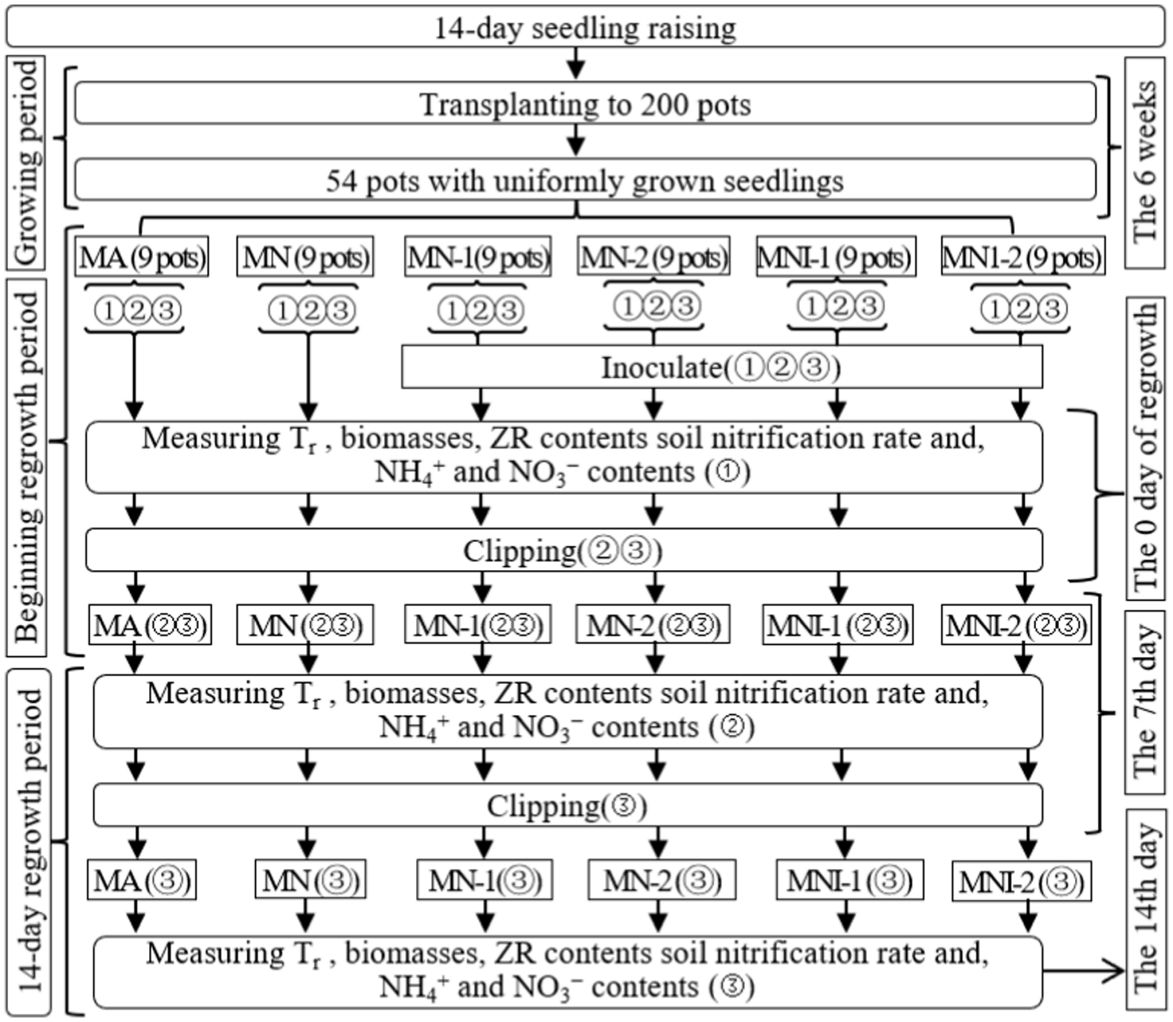
Schematic diagram for the design of experiment-2. MA, MN, MNI1, MNI2, MI1, and MI2 represent adding 100 mL liquid medium without S2_8_1, adding 35 mmol L^−1^ NO_3_^−^, adding 50 mL liquid medium with S2_8_1, adding 100 mL liquid medium with S2_8_1, adding 35 mmol L^−1^ NO_3_^−^ and 50 mL liquid medium with S2_8_1, adding 35 mmol L^−1^ NO_3_^−^ and 100 mL liquid medium with S2_8_1, respectively. “①,” “②,” and “③” show the first, second, and third subgroups of each treatment, respectively. The 7th and 14th days refer to the 14 days regrowth period. All indexes refer to all the indexes that were measured in the present study. *T*_r_ represents the transpiration rate, respectively.

In Exp-2, the 54 selected pots were divided into six groups, each with nine pots corresponding to the study’ treatments. These treatments included: (1) regrowth with 100 mL liquid medium without S2_8_1 (MA), (2) regrowth with 35 mmol·L^−1^ NO_3_^−^ (MN), (3) regrowth with 50 mL liquid medium with S2_8_1 (MN-1), (4) regrowth with 100 mL liquid medium with S2_8_1 (MN-2), (5) regrowth with 35 mmol·L^−1^ NO_3_^−^ and 50 mL liquid medium with S2_8_1 (MNI-1), (6) regrowth with 35 mmol·L^−1^ NO_3_^−^ and 100 mL liquid medium with S2_8_1 (MNI-2). Figure 2 clearly displays the trial timeline, treatment configurations, and the associated parameters measured.

The liquid mediums without or with AOB (216,090 cfu mL^−1^) were added at the beginning of the regrowth period. The initial addition of NO_3_^−^ solution occurred at the start of the regrowth period, with a volume of 200 mL. Throughout the regrowth period, additional NO_3_^−^ solution was administered to maintain the soil water content at 65–70% of the field capacity in the MN, MNI-1, and MNI-2 treatments. Similarly, water was added to the MA, MJ1, and MJ2 treatments to sustain the soil water content at 65–70% of the field capacity during the regrowth period. The methods of adding NO_3_^−^ solution or water remained consistent with those used in Exp-1.

In both Exp-1 and Exp-2, the nine pots in each treatment were further divided into three subgroups, with three pots in each subgroup serving as replicates for each measurement. The three subgroups within each treatment were randomly labeled as subgroups 1, 2, and 3.

### 2.2. Measurement process

At the commencement of the 14 days regrowth period, specifically on day 0, subgroup 1 of each treatment underwent a series of four steps. Firstly, their transpiration rate (*T*_r_) was measured. Subsequently, the plants were clipped to a height of 5 cm, and the freshly clipped material was immediately weighed to determine its fresh weight. Additionally, 0.5 g of fresh clippings was sampled to measure the concentrations of zeatin riboside (ZR) in the leaves. The remaining leaf samples were dried for 72 h at 65°C to determine the dry matter content, calculated by dividing the dry weight by the fresh weight. The clipping biomass was obtained by multiplying the leaf dry matter content by the clipping fresh weight.

Following this, xylem sap was collected using the methods described by Qin and Wang (2020). After clipping the leaf blades of Italian ryegrass, the wounds were promptly covered with 1.0 g of absorbent cotton for 12 h to absorb the xylem sap. The absorbent cotton is wrapped in a plastic film to prevent moisture evaporation. The volume of xylem sap was determined by dividing the increase in cotton weight by 1 g cm^−3^. The rate of xylem sap transport from roots to leaves in the darkness was calculated as the volume of xylem sap divided by 12 h. The cotton was then compressed to extract the sap, which was collected for measuring the concentrations of ZR.

Third, intact roots and soils were carefully extracted from the pots to collect rhizosphere soil samples in subgroup 1 of each treatment. A clean brush was used to gently remove soil particles from all the roots, repeating the process 5–10 times until small soil particles were dislodged. Each collection of soil particles was kept separate to avoid mixing. The soil in close proximity to the roots was designated as rhizosphere soil. The collected rhizosphere soil was then used to measure soil nitrification rate, as well as the concentrations of NH_4_^+^ and NO_3_^−^.

After completing the three-step measurements for subgroup 1 in each treatment as described above, plants in subgroups 2 and 3 were trimmed to a height of 5 cm. They were then given 7 days to regrow. On the 7th day, following the same procedure as subgroup 1 on day 0, the three steps were repeated for subgroup 2. At the same time, subgroup 3 was also clipped to 5 cm and allowed to regrow for another 7 days on day 7. On day 14, the three steps were similarly carried out for subgroup 3. Therefore, each treatment included two clippings on days 0 and 7 within a 14 days period. The 7 days regrowth period was chosen as it is likely to reveal discernible differences in regrowth.

### 2.3. Measurements and data analysis

#### 2.3.1. Biomass, transpiration rate, and soil nitrification

The biomass resulting from clipping in subgroups 2 and 3 refers to the regrown leaves’ biomass for each treatment on day 7 after the first and second clippings, respectively. In subgroup 1, the clipping biomass represents the biomass of the leaves above the stem height of 5 cm before defoliation. To measure the *T*_r_, an LI-6400 device was used between 11:00 am and 12:00 pm under specific experimental conditions, including a light intensity of 1,000 μmol m^−2^ s^−1^, temperature of 28°C, and carbon dioxide concentration ranging from 400 to 420 ppm. Given the narrow leaf blades of Italian ryegrass, a single leaf was insufficient to cover the leaf chamber of the photosynthesis equipment. Consequently, 2–3 leaves were tightly overlapped to increase the leaf area and adequately cover the chamber.

The pot walls were cut open to take the soil and root out as a whole. Then, the soils were meticulously removed from the entire root by patting. This patting process was repeated several times until small soil particles were removed from the roots. These small soil particles were collected, forming the rhizosphere soil used in this study. Soil NH_4_^+^ and NO_3_^−^ contents were measured using the indophenol blue method and phenol disulfonic acid colorimetry, respectively (Lu, 1999). Soil samples whose moisture was maintained at 60% field capacity were cultured for 7 days at 25°C to determine the soil net nitrification rate. The differences in soil NO_3_^−^ content before and after culturing were divided by 7 days to obtain the soil net nitrification rate of each day.

#### 2.3.2. Zeatin riboside

The ZR is a derivative of zeatin and is a structurally stable cytokinin that functions in hormone signaling and metabolic pathways. The ZR content can be reliably detected in the xylem sap and in leaves. Moreover, there’s a significant positive correlation between leaf ZR content and regenerative leaf biomass in Italian ryegrass, making it an essential indicator for cytokinin levels measurement (Wang et al., 2012).

For ZR concentration measurement, sap from each sample was pooled in 5 mL centrifuge tubes and subsequently injected into a solid-phase extraction C-18 column for filtration. After that, it was dried using a nitrogen blow-dryer, followed by extraction using 80% methanol containing 1 mmol L^−1^ di-tert-butyl-4-methylphenol. Similar procedures were followed for leaf extracts. Residues from both the leaf and xylem sap extracts were dissolved in 0.01 mol L^−1^ phosphate buffer solution (pH 7.4) and subjected to an enzyme-linked immunosorbent assay (ELISA) to determine the concentration of ZR, as described by Qin and Wang (2020). The test kits for zeatin riboside were produced at the Phytohormone Research Institute of China Agricultural University.

The ELISA was performed in a 96-well microtitration plate. Each well was coated with 100 μL of the coating buffer that contained 0.25 μg mL^−1^ hormone antigens. After washing four times, each well was received 50 μL of either plany sample extract or ZR standards and 50 μL of 20 μg mL^−1^ antibodies against ZR. The plates were incubated for 3 h at 28°C to determine ZR concentrations. After further washing, each well was treated with 100 μL of 1.25 μg mL^−1^ IgG–horseradish peroxidase substrate and incubated for 1 h at 30°C. The plates underwent five rinses and were then provided with 100 μL of color-appearing solution. Color development in each well was detected using an ELISA Reader at an optical density of A490.

The leaf ZR concentrations were determined by dividing its amounts by the weight of the leaf sample. Similarly, the ZR concentrations of xylem sap (C_ZR_) were obtained by dividing its amounts by the volume of the sap. The transport rates of zeatin riboside from roots to leaves were calculated separately for darkness (B_ZR_) and light (L_ZR_). B_ZR_ and L_ZR_ was calculated, respectively, using Formulas (2) and (3):


(2) $$B_{ZR}=C_{zr}\times X_{r}$$

(3) $$L_{ZR}=C_{zr}\times T_{r}\times M\times SLA$$

where *X*_r_ represents the rate of xylem sap transport in darkness, *T*_r_ is the transpiration rate, M is the regrown leaves biomass, and SLA is the specific leaf area, which refers to the ratio of leaf surface are to leaf dry weight, and is determined in advance.

#### 2.3.3. Data analysis

All values given in the figure are average values. The general linear model in SPSS 23 was used to conduct one-way analysis of variance followed by Dunnett test at the 0.05 probability level.

## 3. Results

### 3.1. Exp-1

**Figure 3:**
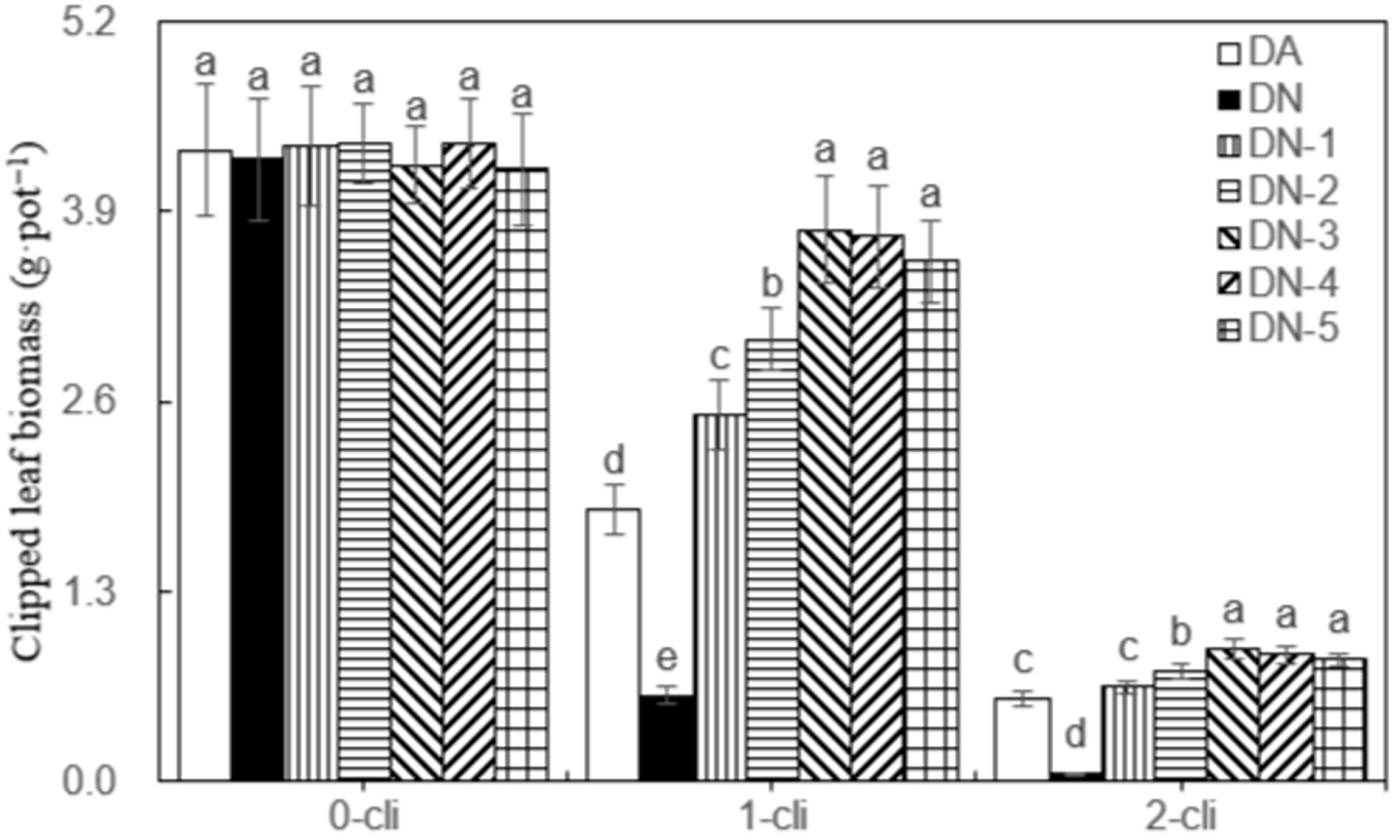
Clipped leaf biomass in different treatments of experiment-1. Different lowercase letters in the figure represent significant differences (*p* < 0.05). “0-cli,” “1-cli” and “2-cli” mean days 0, 7 and 14 after defoliation, respectively. DA, DN, DN1, DN2, DN3, DN4, and DN5 represent conventional water supply treatment, adding DMPP, adding DMPP and 10 mmol L^−1^ NO_3_^−^-N, adding DMPP and 20 mmol L^−1^ NO_3_^−^-N, adding DMPP and 30 mmol L^−1^ NO_3_^−^-N, adding DMPP and 40 mmol L^−1^ NO_3_^−^-N and adding DMPP and 50 mmol L^−1^ NO_3_^−^-N, respectively.

**Figure 4:**
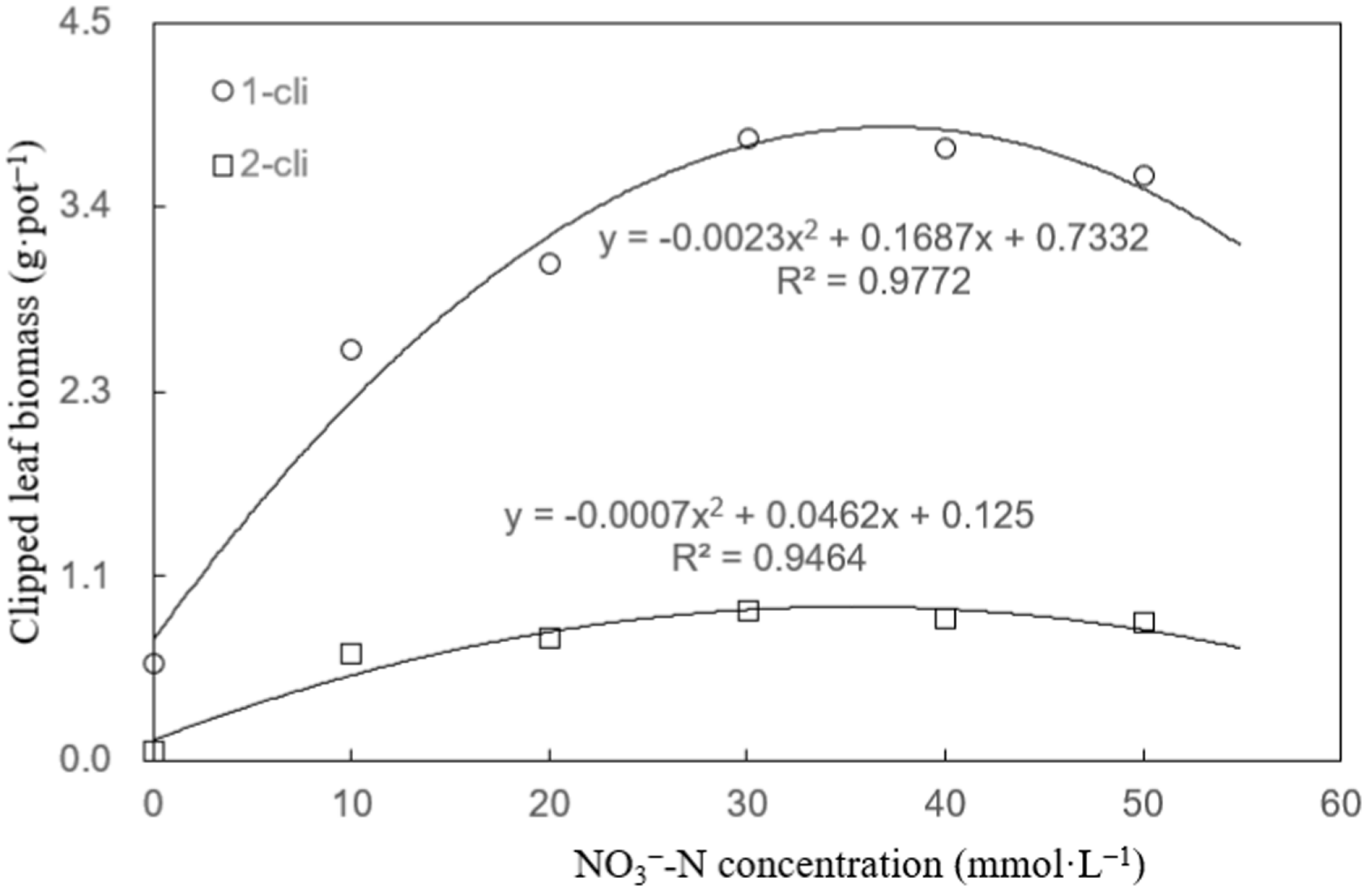
Clipped leaf biomass regression analysis plot of experiment-1. “1-cli” and “2-cli” mean days 7 and 14 after defoliation, respectively.

Figure 3 illustrates that DMPP significantly restricted the regrowth of Italian ryegrass, given that a noticeable decrease in regrown leaf biomass was observed in the DN treatment compared to the DA treatment on days 7 after the first and second defoliations. However, the negative impact of DPMM on regrowth was reversed when NO_3_^−^ was added. Treatments with NO_3_^−^, such as DN-1, DN-2, DN-3, DN-4, and DN-5, exhibited significantly higher regrown leaf biomasses compared to DA and DN treatments. Regression analysis in Figure 4 indicates that, the NO_3_^−^ of 33.2 mmol L^−1^ concentration, namely between 30–40 mmol L^−1^, could cause the optimal increase effect on Italian ryegrass regrowth.

**Figure 5:**
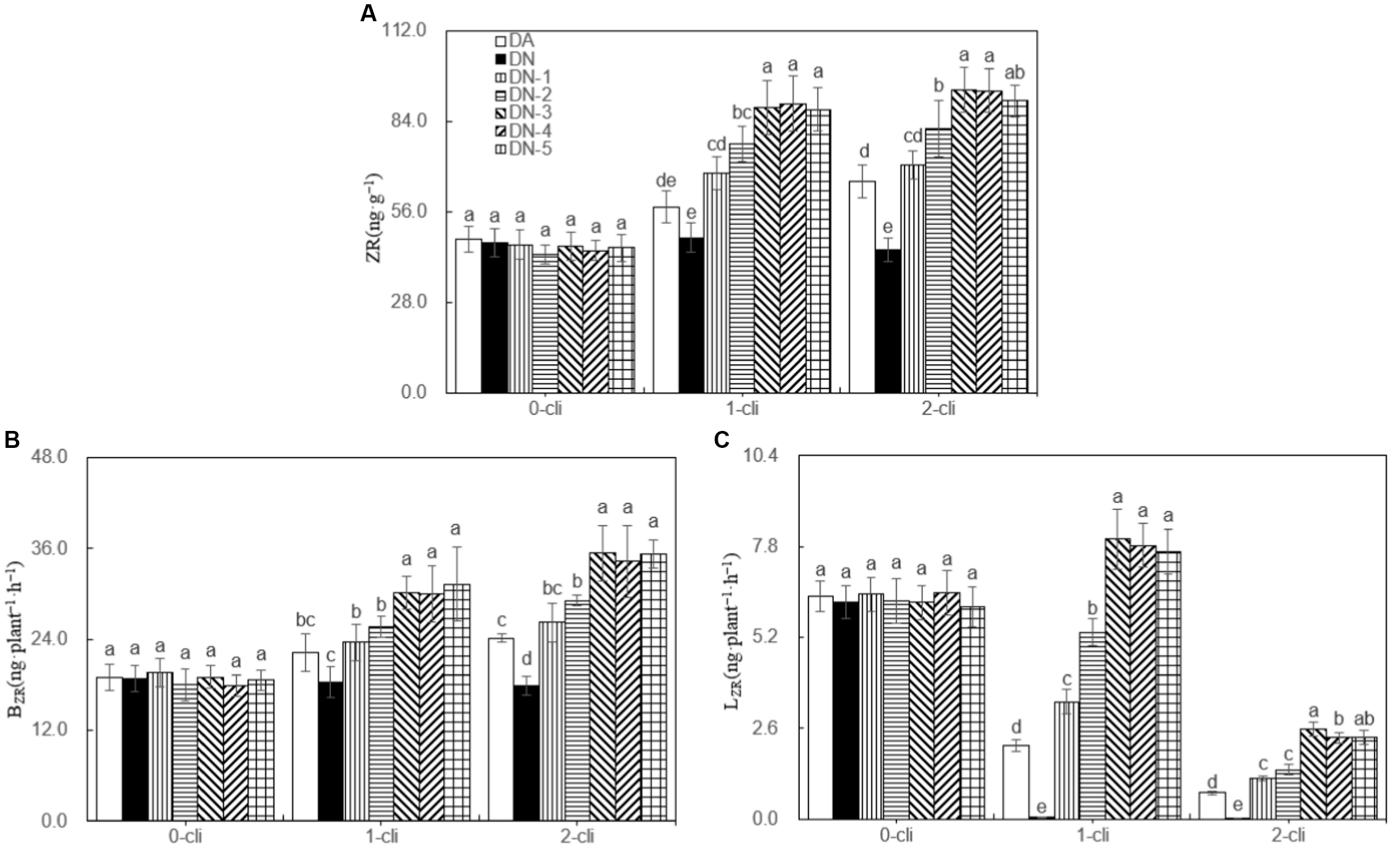
ZR, B_ZR_, and L_ZR_ in different treatments of experiment-1. **(A)** ZR represents cytokinin, **(B)** B_ZR_ represents the transport rates of zeatin riboside from roots to leaves were calculated separately for darkness, **(C)** L_ZR_ represents the transport rates of zeatin riboside from roots to leaves were calculated separately for darkness. Different lowercase letters in the figure represent significant differences (*p* < 0.05). “0-cli,” “1-cli” and “2-cli” mean days 0, 7, and 14 after defoliation, respectively. DA, DN, DN1, DN2, DN3, DN4, and DN5 represent conventional water supply treatment, adding DMPP, adding DMPP and 10 mmol L^−1^ NO_3_^−^-N, adding DMPP and 20 mmol L^−1^ NO_3_^−^-N, adding DMPP and 30 mmol L^−1^ NO_3_^−^-N, adding DMPP and 40 mmol L^−1^ NO_3_^−^-N and adding DMPP and 50 mmol L^−1^ NO_3_^−^-N, respectively.

**Figure 6:**
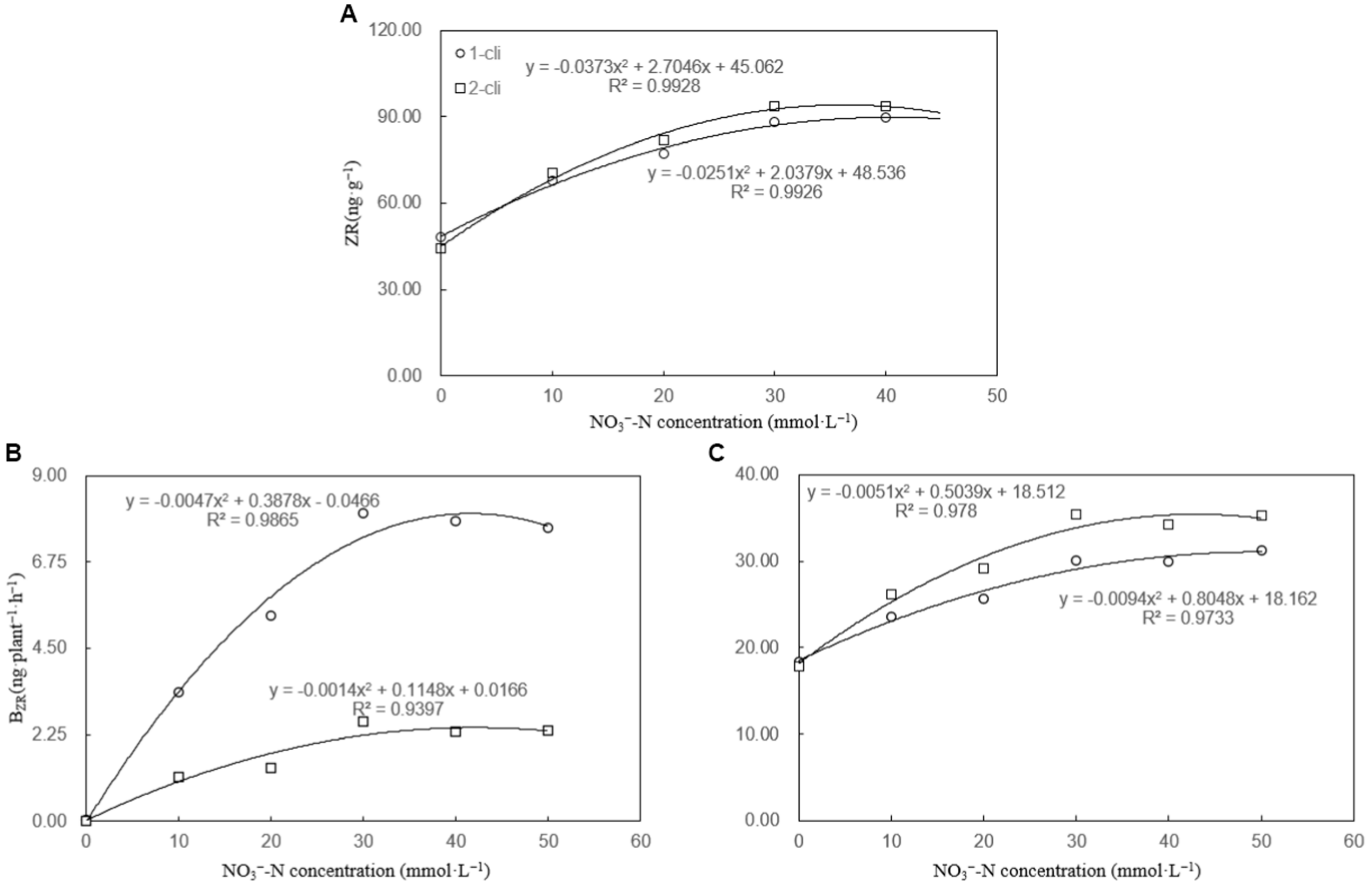
ZR, B_ZR_, and L_ZR_ regression analysis plot of experiment-1. **(A)** ZR regression analysis plot, **(B)** B_ZR_ regression analysis plot, and **(C)** L_ZR_ regression analysis plot. “1-cli” and “2-cli” mean days 7 and 14 after defoliation, respectively.

The leaf ZR content, B_ZR_, and L_ZR_ were significantly higher in DA compared to DN, as well as in DN-1, DN-2, DN-3, DN-4, and DN-5 compared to DA, on day 7 after the first and second defoliations (Figure 5). These results, considering ZR as the assay index of cytokinin, suggest that DMPP reduced the transport of cytokinin from roots to leaves, leading to reduced leaf cytokinin content in Italian ryegrass. However, the addition of NO_3_^−^ reversed the negative impact of DPMM on them. Regression analysis (Figure 6) revealed that NO_3_^−^ concentrations ranging from 30–40 mmol·L^−1^ had the optimal stimulatory effect on leaf ZR content, B_ZR_, and L_ZR_. The optimal concentrations were 33.2, 32.5, and 38.6 mmol·L^−1^, respectively.

### 3.2. Exp-2

#### 3.2.1. Biomass

**Figure 7:**
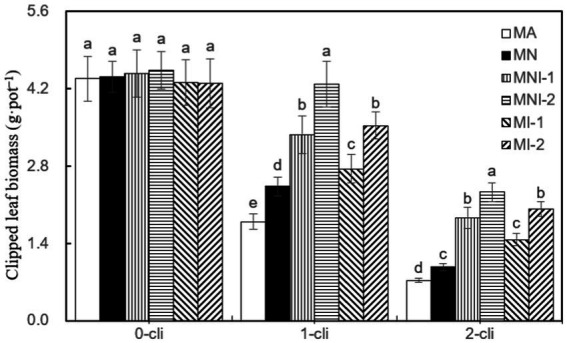
Clipped leaf biomass in different treatments of experiment-2. Different lowercase letters in the figure represent significant differences (*p* < 0.05). “0-cli,” “1-cli”, and “2-cli” mean days 0, 7, and 14 after defoliation, respectively. MA, MN, MNI1, MNI2, MI1, and MI2 represent adding 100 mL liquid medium without S2_8_1, adding 35 mmol L^−1^ NO_3_^−^, adding 50 mL liquid medium with S2_8_1, adding 100 mL liquid medium with S2_8_1, adding 35 mmol L^−1^ NO_3_^−^ and 50 mL liquid medium with S2_8_1, adding 35 mmol L^−1^ NO_3_^−^ and 100 mL liquid medium with S2_8_1, respectively.

According to Figure 7, the regrown leaf biomass was significantly lower in MA treatment compared to all other treatments on day 7 after the first defoliation. Similarly, it was significantly lower in MA compared to MNI-1 and MNI-2 on day 7 after the second defoliation. Thus, the addition of NO_3_^−^ and S2_8_1 inoculation, either individually or in combination, all played a significant role in increasing regrowth. Notably, the S2_8_1 exhibited a more pronounced promoting effect than the NO_3_^−^ addition, as significantly higher regrown leaf biomass occurred in MI-1 and MI-2 compared to MN on the 7th day after both the first and second defoliations. Additionally, significantly higher regrowth leaf biomass was noted in MNI-1 and MNI-2 compared to MN, in MI-2 compared to MI-1, and in MNI-2 compared to MNI-1 on day 7 after either the first or second defoliations. These findings highlight the synergistic effect of HAOB stain with NO_3_^−^, where higher HAOB stain concentration resulted in greater promotion of regrowth.

#### 3.2.2. Plant zeatin riboside

**Figure 8:**
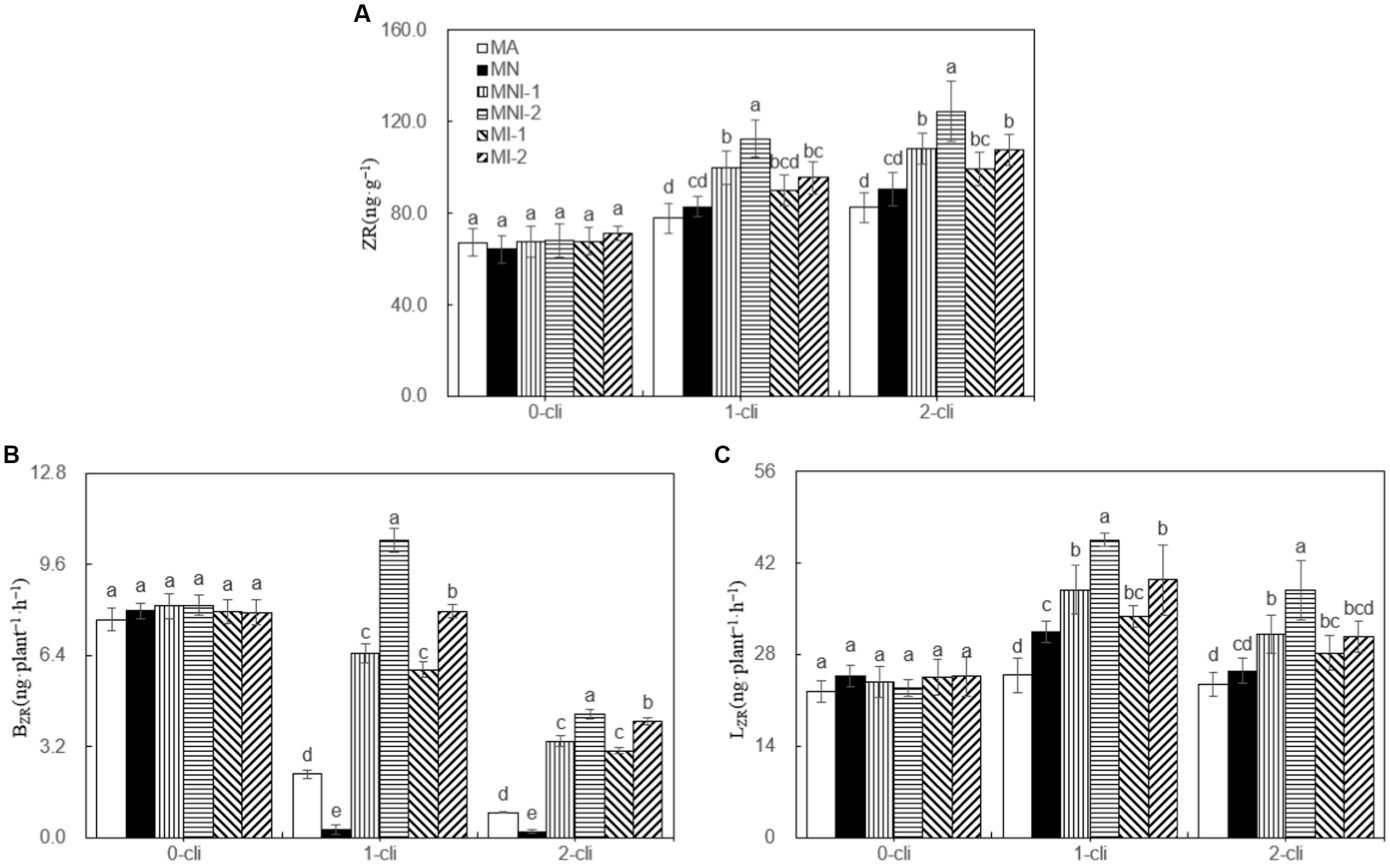
ZR, B_ZR_, and L_ZR_ in different treatments of experiment-2. **(A)** ZR represents cytokinin, **(B)** B_ZR_ represents the transport rates of zeatin riboside from roots to leaves were calculated separately for darkness, **(C)** L_ZR_ represents the transport rates of zeatin riboside from roots to leaves were calculated separately for light. Different lowercase letters in the figure represent significant differences (*p* < 0.05). “0-cli,” “1-cli”, and “2-cli” mean days 0, 7, and 14 after defoliation, respectively. MA, MN, MNI1, MNI2, MI1, and MI2 represent adding 100 mL liquid medium without S2_8_1, adding 35 mmol L^−1^ NO_3_^−^, adding 50 mL liquid medium with S2_8_1, adding 100 mL liquid medium with S2_8_1, adding 35 mmol L^−1^ NO_3_^−^ and 50 mL liquid medium with S2_8_1, adding 35 mmol L^−1^ NO_3_^−^, and 100 mL liquid medium with S2_8_1, respectively.

On days 7 after the first or second defoliations, the leaf ZR content, B_ZR_, and L_ZR_ were significantly lower in the MA treatment compared to all other treatments (Figure 8). This indicates that the NO_3_^−^ addition, HAOB strain inoculation, or their combination all increased cytokinin contents in leaves and its transport from roots. Notably, the HAOB strain had a stronger effect than NO_3_^−^, as MI-1 and MI-2 treatments had significantly higher leaf ZR content, B_ZR_, and L_ZR_ compared to MN on day 7 after the first or second defoliations. Combining the HAOB strain with NO_3_^−^ had a greater promotional effect compared to the HAOB strain alone, as evidenced by higher leaf ZR content, B_ZR_, and L_ZR_ in MNI-2 compared to MI-2 on day 7 after each defoliation. Furthermore, a higher concentration of the HAOB strain played a greater role, with MNI-2 showing higher the leaf ZR content, B_ZR_, and L_ZR_ than MNI-1 during the regrowth period.

#### 3.2.3. Soil nitrification rate, soil ammonium, and nitrate nitrogen

**Figure 9:**
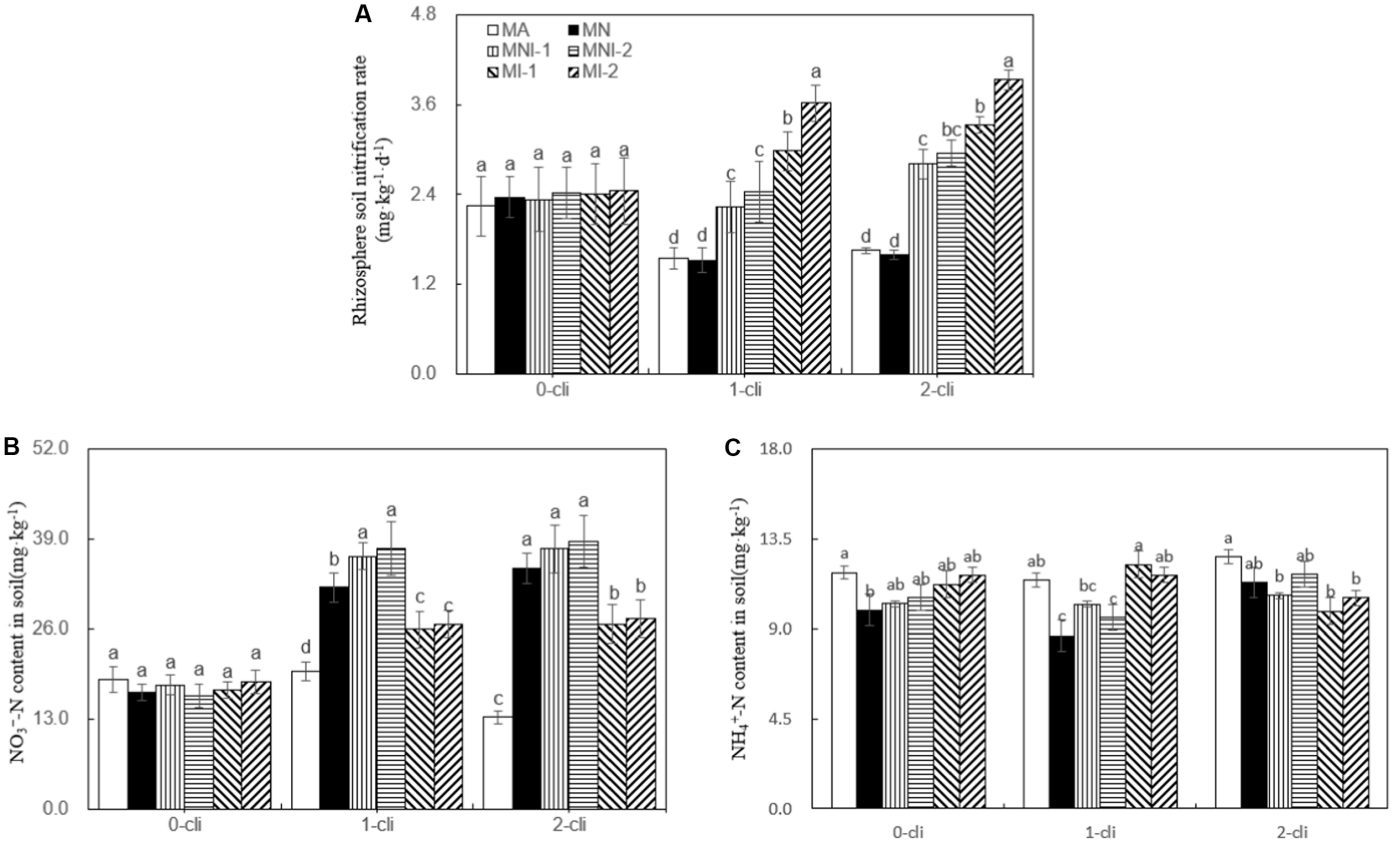
Rhizosphere soil nitrification rates, NO_3_^−^-N contents and NH_4_^+^-N contents in soil in different treatments of experiment-2. **(A)** Rhizosphere soil nitrification rates in soil in different treatments, **(B)** Rhizosphere soil NO_3_^−^-N contents in different treatments, **(C)** Rhizosphere soil NH_4_^+^-N contents in different treatments. Different lowercase letters in the figure represent significant differences (*p* < 0.05). “0-cli,” “1-cli”, and “2-cli” mean days 0, 7, and 14 after defoliation, respectively. MA, MN, MNI1, MNI2, MI1, and MI2 represent adding 100 mL liquid medium without S2_8_1, adding 35 mmol L^−1^ NO_3_^−^, adding 50 mL liquid medium with S2_8_1, adding 100 mL liquid medium with S2_8_1, adding 35 mmol L^−1^ NO_3_^−^ and 50 mL liquid medium with S2_8_1, adding 35 mmol L^−1^ NO_3_^−^ and 100 mL liquid medium with S2_8_1, respectively.

As depicted by Figure 9, on day 7 after the first or second defoliations, significant increases in rhizosphere soil nitrification rates were observed in the treatments of MI-1 and MI-2 compared with MA and MN treatments, but no such significant differences were found between MA and MN treatments. This indicates that S2_8_1 inoculation positively influenced the nitrification rate, while NO_3_^−^ addition had no such effect. Moreover, the rhizosphere soil nitrification rates were significantly higher in MI-1 than in MNI-1 and in MI-2 than in MNI-2 on day 7 after each defoliation, suggesting a potential negative influence of NO_3_^−^ addition on soil nitrification under S2_8_1 inoculation. During the rewatering period, the addition of NO_3_^−^ resulted in significantly higher NO_3_^−^ contents in rhizosphere soils in MN than in MA, in MNI-1 than in MI-1, and in MNI-2 than in MI-2. However, it had little influence on rhizosphere soil NH_4_^+^ contents.

## 4. Discussion

### 4.1. Optimal NO_3_^−^ concentration

In general, plant root cytokinins can be transported to aboveground stems and leaves through xylem sap (Zaicovski et al., 2008; Wang et al., 2016). Furthermore, the root tip’s apical meristem, pivotal for cytokinin synthesis and nutrient absorption, demonstrates an increased synthesis of cytokinins in response to NO_3_^−^ stimulation in various plants (Cortleven et al., 2019; Oldroyd and Leyser, 2020). This effect primarily arises from the roots’ ability to perceive soil NO_3_^−^ and subsequently transmit this signal via cytokinins to the leaves. In the Exp-1, B_ZR_ and L_ZR_ values exhibited an upward trend with increasing NO_3_^−^ concentrations, ranging from 0 to 30 mmol L^−1^, and then remained stable between 30 to 50 mmol L^−1^. Regression analysis highlighted that the most effective promotion of B_ZR_ and L_ZR_ by NO_3_^−^ occurred within the 30–40 mmol L^−1^ concentration range. Consequently, this specific NO_3_^−^ concentration range yielded the highest B_ZR_ and L_ZR_ values, resulting in enhanced cytokinin synthesis and transport to leaves, leading to elevated leaf cytokinin concentrations.

The constrained ability of roots to sense NO_3_^−^ signals likely governs the phenomenon of a peak in cytokinin transmission to the leaves observed in this study. As NO_3_^−^ concentrations escalate from 0 to 30 mmol L^−1^, they increase the presence of NO_3_^−^ in the soil, heightening the likelihood of interaction with the roots of Italian ryegrass. This, in turn, intensifies the signal of root-derived cytokinin, leading to an accelerated rate of cytokinin transmission to the leaves, as indicated by the progressively rising B_ZR_ and L_ZR_ values. However, when NO_3_^−^ concentrations exceed a specific threshold, surplus NO_3_^−^ in the soil results in congestion, reducing the potential for enhanced NO_3_^−^-root contact due to the consistent root surface area. This leads to peak values of B_ZR_ and L_ZR_ at NO_3_^−^ concentrations ranging from 30 to 40 mmol L^−1^.

The study revealed that specific NO_3_^−^ concentrations, namely 33.2, 33.5, 32.5, and 38.6 mmol L^−1^, had the most favorable impact, respectively, on leaf regrowth biomass, BZR, LZR, and leaf cytokinin content. Therefore, the selection of 35 mmol L^−1^ NO_3_^−^ concentrations in this study closely aligns with the optimal increase effect on the cytokinin transport system from roots to leaves and its subsequent effect on the regrowth process.

### 4.2. Dual roles of outside and inside soils within the rhizosphere microenvironment

Considering that ammonia-oxidizing bacteria are the primary bacteria involved in soil nitrification, the HAOB strain inoculation treatments of the Exp-2 significantly increased rhizosphere soil nitrification rates by an average of 94% during the regrowth period compared to non-inoculation treatments. This higher nitrification rate led to a continuous release of more NO_3_^−^ from the soil during the rewatering period in HAOB strain inoculation treatments of MI-1 and MI-2 compared with the MA treatment without inoculation, further enhancing the stimulation of NO_3_^−^ on the roots. As a result, there was an average of 1.61 times higher rhizosphere soil NO_3_^−^ content in MI-1 and MI-2 compared with the MA treatment, resulting in more root cytokinins being synthesized and continuously delivered. This led to a 20% higher leaf cytokinin content in MI-1 and MI-2 compared to the MA treatment.

Based on the analysis in “4.1 Optimal NO_3_^−^ concentration,” adding a 35 mmol L^−1^ NO_3_^−^ solution showed the potential to achieve the optimal increase effect on the cytokinin transport from roots to leaves in MN, MI-1, and MI-2 treatments of the Exp-2. Therefore, the higher leaf cytokinin contents in MI-1 and MI-2 compared with MN could not solely be attributed to the effect of NO_3_^−^ released from the soil due to S2_8_1-mediated nitrification. It is plausible that another mechanism exists, potentially involving S2_8_1, to increase root cytokinin synthesis apart from nitrification. It is likely that S2_8_1 caused higher B_ZR_ and L_ZR_ levels in MI-1 and MI-2 during the regrowth period through its “soil-outside-role.” This role refers to S2_8_1 operate outside the rhizosphere soil within rhizosphere microenvironment, possibly enhancing cytokinin transport from roots to leaves within the internal root tissues. Thus, the present study suggests that S2_8_1 played dual roles in increasing root cytokinin synthesis in both the inside and outside rhizosphere soils.

In the Exp-2, besides NO_3_^−^, S2_8_1 still enhanced cytokinin delivery from roots to leaves, as observed in the MNI-2 treatment where B_ZR_ and L_ZR_ increased by over 20% compared to MN and MI-2 treatments during the regrowth period. Consequently, MNI-2 exhibited 17 and 37% higher leaf cytokinin contents than MI-2 and MN, respectively. This highlights the relatively independent process of S2_8_1’s soil-outside-role in cytokinin delivery, which is minimally influenced by its “soil-inside-role” that refers to S2_8_1 operating inside the rhizosphere soil. Wang et al. (2021, 2022) also reported that the HAOB strain had a stronger impact on cytokinin delivery and leaf cytokinin content in corn compared to the addition of NO_3_^−^ to roots.

The inoculation size of the MNI-1 was only half of that in the MNI-2, resulting in fewer chances for S2_8_1 to reach roots. As a result, the MNI-2 exhibited 50, 21, and 14% higher L_ZR_, B_ZR_, and leaf cytokinin contents than MNI-1 during the regrowth period, respectively, once again confirming the soil-outside-role of S2_8_1.

### 4.3. Significance of the dual roles

The higher leaf cytokinin contents in MN compared with MA resulted in a 35% increase in leaf regrowth biomass during the regrowth period, indicating the significant impact of NO3^−^ in inducing cytokinins on Italian ryegrass regrowth. Similarly, the elevated leaf cytokinin contents in MNI-2 compared with MN facilitated a remarkable 94% increase in the regrowth biomass, highlighting the significant impact of S2_8_1’s soil-outside-role on Italian ryegrass regrowth. Furthermore, S2_8_1’s soil-outside-role, coupled with its soil-inside-role in increasing NO3^−^ release from soils, synergistically enhances cytokinin delivery from roots to leaves, contributing to a 120% increase in leaf regrowth biomass in MI-2 compared with MA on day 14 after defoliation. Therefore, the dual roles of S2_8_1, both inside and outside the rhizosphere soil within the rhizosphere microenvironment, play a crucial role in Italian ryegrass regrowth and hold great potential for enhancing the growth of other crops, such as wheat, corn, rice, and more.

The ammonia-oxidizing bacteria encompasses a diverse range of bacteria, including both autotrophic and heterotrophic AOBs. Previous studies primarily focused on their vital role in nitrification processes within aquatic and soil environments (Taylor et al., 2009; Kouki et al., 2011; Matsuno et al., 2013; Soliman and Eldyasti, 2018; Fang et al., 2019). A limited body of research has revealed that HAOB strains promote soil nitrification in the rhizosphere, leading to the release of nitrate nitrogen from the soil (Wu et al., 2022, 2023). However, our study introduces a novel perspective by exploring their role in enhancing plant growth. This effect results from the combined influence of HAOB’s soil-inside-role and soil-outside-role within the rhizosphere microenvironment on the transport of cytokinins from roots to leaves, with a special emphasis on the significant role of the soil-outside-role.

**Figure 10:**
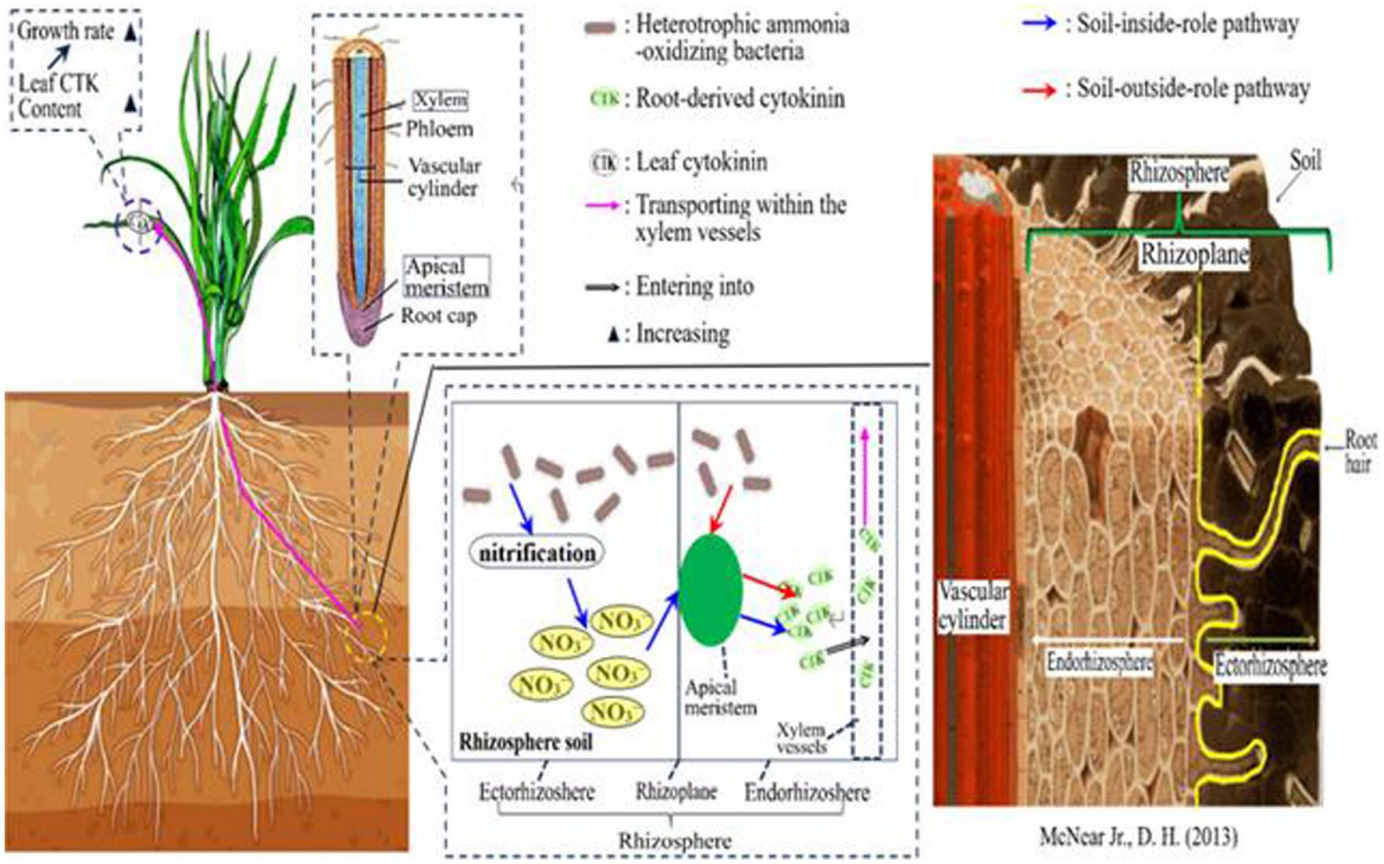
Mechanistic diagram of the dual roles of HAOB.

Nonetheless, the specific mechanism underlying the “soil-outside-role” of HAOB strains is not yet fully understood. To address this, our study constructs a mechanistic diagram to comprehensively elucidate the roles of both the ‘soil-inside-role’ and, particularly, the ‘soil-outside-role.’ In the mechanistic diagram, we propose that HAOB strains may directly enter the roots to enhance cytokinin synthesis, as depicted in Figure 10. However, this hypothesis requires further investigation for validation. If confirmed, it would significantly advance our understanding of HAOB’s impact on crop growth, thereby enhancing their potential applications in agriculture.

To further advance our understanding and address potential limitations of this study, future research directions may include exploring the long-term effects of HAOB inoculation on both soil health and sustained plant growth. Investigating the extended-term impacts can provide valuable insights into the durability and stability of the observed enhancements, contributing to more comprehensive knowledge in this field.

## 5. Conclusion

In this study, we made a significant discovery regarding the role of different concentrations of NO_3_^−^ in promoting regrowth of Italian ryegrass. We found that an optimal concentration of NO_3_^−^, between 30–40 mmol L^−1^, had the highest positive effect on both regrowth biomass and leaf cytokinin content. Our research also showed that the inoculation of S2_8_1 played a crucial role in promoting cytokinin delivery from the roots to the leaves, benefiting the regrowth. This effect was attributed to two mechanisms: the soil-inside-role and soil-outside-role. The soil-inside-role was associated with increased rhizosphere soil nitrification rates, leading to more NO_3_^−^ release from the soil and enhancing cytokinin delivery. Furthermore, S2_8_1 exhibited a soil-outside-role, independently boosting cytokinin delivery beyond the influence of NO_3_^−^ caused by the soil-inside-role. The combined effects of these roles significantly increased leaf cytokinin content, further supporting the regrowth of Italian ryegrass.

## Data availability statement

The original contributions presented in the study are included in online repositories, accession numbers can be found in the article/supplementary materials, further inquiries can be directed to the corresponding author/s.

## Author contributions

X-LW: Project administration, Supervision, Writing – original draft. Z-QS: Writing – review and editing. HY: Writing – original draft, Writing – review and editing. LQ: Validation, Writing – review and editing. WL: Validation, Data curation, Writing – review and editing. JS: Formal analysis, Data curation, Writing – review and editing. PS: Formal analysis, Data curation, Writing – review and editing.

## Funding

The author(s) declare financial support was received for the research, authorship, and/or publication of this article. This work was supported by the National Natural Science Foundation of China (U1304326), the Excellent Youth Foundation of Henan Scientific Committee (174100510004), and the Henan Natural Science Foundation (162300410070).

## Conflict of interest

The authors declare that the research was conducted in the absence of any commercial or financial relationships that could be construed as a potential conflict of interest.

## Publisher’s note

All claims expressed in this article are solely those of the authors and do not necessarily represent those of their affiliated organizations, or those of the publisher, the editors and the reviewers. Any product that may be evaluated in this article, or claim that may be made by its manufacturer, is not guaranteed or endorsed by the publisher.
